# The Influence of End-of-Day Blue Light on the Growth, Photosynthetic, and Metabolic Parameters of Lettuce at Different Development Stages

**DOI:** 10.3390/plants11202798

**Published:** 2022-10-21

**Authors:** Viktorija Vaštakaitė-Kairienė, Giedrė Samuolienė, Vaidevutis Šveikauskas, Kristina Laužikė, Sigita Jurkonienė

**Affiliations:** 1 Lithuanian Research Centre for Agriculture and Forestry, Institute of Horticulture, Kaunas Street 30, 54333 Babtai, Lithuania; 2 Nature Research Centre, Laboratory of Plant Physiology, Akademijos Street 2, 08412 Vilnius, Lithuania

**Keywords:** controlled environment agriculture, light-emitting diodes, physiological response, metabolites

## Abstract

This study evaluates the effect of end-of-day blue (EOD B) light on the physiological response of lettuce (*Lactuca sativa*, Lobjoits Green Cos) at different phenological development stages. Plants were grown in a controlled environment growth chamber (day/night temperature 21 ± 2 °C; relative air humidity 60 ± 5%) under the light of light-emitting diodes (LEDs) consisting of 5% blue (B; 450 nm), 85% red (R; 660 nm), and 10% green (G; 530 nm) photosynthetic photon flux density (PPFD) at 200 µmol m^−2^ s^−1^ for 16 h d^−1^ (BRG, control) for 8, 15, and 25 days (BBCH 12, BBCH 14, and BBCH 18, respectively). For the EOD B treatments, lettuce plants were additionally illuminated with 100% of B light at 30 and 60 µmol m^−2^ s^−1^ PPFD for 4 h d^−1^ (B_30_ and B_60_, respectively). The results show that EOD B light caused the elevated shoot elongation of lettuce plants regardless of their growth stages. However, leaf width increased only in more developed lettuce plants (BBCH 18). EOD B light negatively affected the development of new leaves and fresh weight, except for seedlings (BBCH 12). Most photosynthetic and spectral leaf indices also decreased when lettuce was treated with EOD B light, especially under the PPFD level of 60 µmol m^−2^ s^−1^. Moreover, the changes in metabolic parameters such as DPPH free radical activity, free proline content, and H^+^-ATPase activity in lettuce showed a plant response to unfavorable conditions to EOD B light.

## 1. Introduction

Light is the primary source of energy and an environmental signal for healthy plant growth and development [1,2]. The primary vital process in plants, photosynthesis, is driven by the light energy absorbed by photosynthetic receptors chlorophylls and carotenoids in blue (B, 400–500 nm) and red (R, 600–700 nm) spectral regions [3]. Moreover, plants can sense, receive, and transmit light signals through different light-absorbing photoreceptors, and then regulate the working mechanism of corresponding genetic media so that they can complete the corresponding series of reactions under different light signals [4,5,6]. These include red/far-red absorbing phytochromes (PHY), blue/UV-A absorbing cryptochromes (CRY) and phototropins (PHOT), and UV-B absorbing UVR8 [7]. In the last few decades, many researchers globally investigated how specific light wavelengths affect growth, development, and metabolic process in various horticultural plants. Most of the studies were performed in controlled-environment agricultural (CEA) systems, such as greenhouses or growth chambers, where artificial lighting is used to seek year-round production [8,9,10,11,12,13]. Recently, the technology of light-emitting diodes (LEDs) has gained popularity among other artificial lighting sources (fluorescent, high-pressure sodium, metal halide, and incandescent lamps) due to the small size, specific wavelength, low thermal output, adjustable light intensity and quality, and high photoelectric conversion efficiency [14]. LEDs allow for wavelengths to be matched to plant photoreceptors to have optimal production, and influence plant morphology and metabolism [15]. Among the many photophysiological research studies that have been carried out using LEDs as a sole light source in CEA, most attention has been focused on evaluating the effects of different light wavelengths and their ratio on various physiological aspects of horticultural plants during the daily photoperiod [16,17,18,19,20]. However, there was less focus on evaluating the effects of end-of-day (EOD) light on plants. Most EOD studies have focused on PHY-mediated responses to R and far-red (FR) light. EOD FR light is effective nonchemical means of allowing for the transplant propagation industry to produce long hypocotyls for grafting use [21]. EOD FR-treated tomato (*Solanum lycopersicum*) plants grew higher [22]. Liu et al. [23] showed that auxin plays an essential role in the EOD FR light-mediated hypocotyl elongation of pumpkin (*Cucurbita moschata*), shedding light on the mechanisms of EOD FR mediated hypocotyl elongation. The EOD R light led to a higher mass of dill (*Anethum graveolens*) plants. EOD studies that focused on CRY-mediated responses to B light demonstrated contradictory results. For example, dill treated with EOD B light was characterized by the poorest growth rate [24]. On the other hand, low-intensity EOD B light promoted lettuce plant growth by increasing leaf area and shoot fresh weight [25]. Due to such contradictions, more detailed studies are required. 

Lettuce (*Lactuca sativa*) is the most popular leafy vegetable grown indoors and can be consumed at various growth stages raw or cooked. This plant is a source of vitamins A, K, and C, and metabolites such as phenolic compounds related to human health [26]. Seedling production is an essential part of vegetable crop production. High-quality young plants are a valuable product that can improve early crop establishment, increase the quality, uniformity, and yield of the final harvest, and shorten the production time [27]. Recently, more leafy greens have been harvested at the early baby-leaf stage due to good leaf size, color, and texture, and thicker leaves that allow for a longer shelf-life [28]. 

A previous study showed distinct metabolic response of *Brassica* leafy greens to the same LED light conditions in different growth stages, and baby-leaf plants showed higher nutritional value in comparison to microgreens or mature plants [29]. Lettuce plants at the seedling and vegetative stages have different requirements to LED lighting, and the optimal photon distribution of LED lighting should be determined for each stage [30] The growth, photosynthetic, and metabolic parameters of lettuce plants depend on the quantity of B light in the spectrum [31,32,33]. In our study, we evaluated the effects of EOD B light on the parameters of lettuce plants mentioned above. We hypothesized that the physiological response of lettuce plants to EOD B light would depend on their development stage. We also grew plants under two photosynthetic photon flux densities (PPFD) of B light to evaluate differences in the photophysiological responses of lettuce plants. 

## 2. Results

### 2.1. Growth

**Figure 1:**
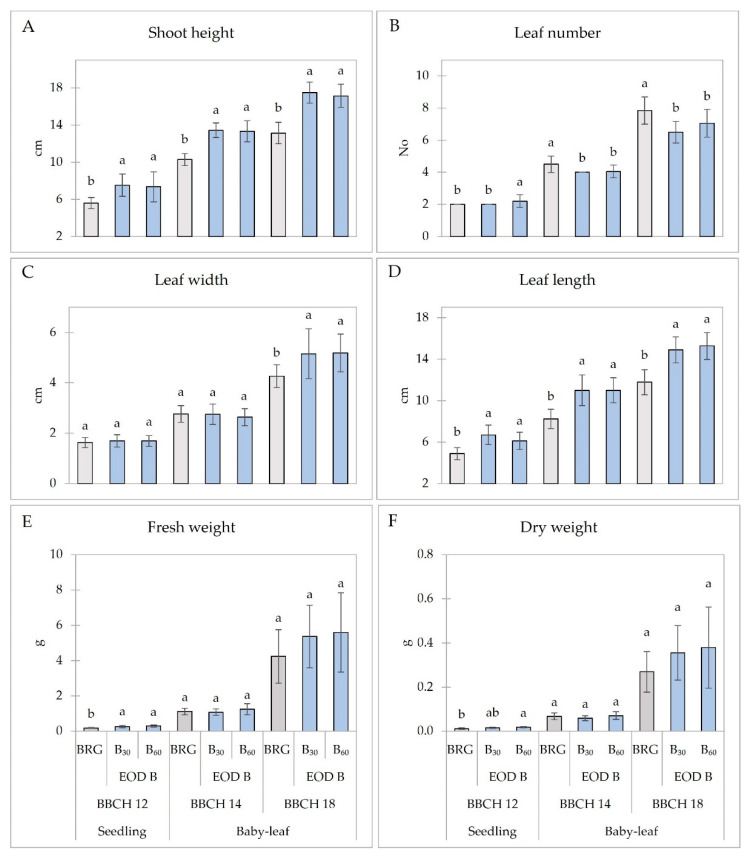
Biometric indices ((**A**) shoot height; (**B**) leaf number; (**C**) leaf width; (**D**) leaf length; (**E**) fresh weight; (**F**) dry weight) of lettuce plants at different development stages. BRG, 5% blue (peak = 450 nm), 85% red (peak = 660 nm), 10% green (peak = 530 nm) for 16 h d^−1^ (Control; from 06:00 to 22:00); B_30_ and B_60_, PPFD of end-of-day lighting with blue (peak = 450 nm) at 30 and 60 µmol m^−2^ s^−1^, respectively; EOD, end-of-day lighting for 4 h d^−1^ (from 22:00 to 02:00). Data are means of two replications with ten samples per replication (n = 20). Means with different letters are significantly different from the control treatment (BRG) at *p* < 0.05.

The additional EOD B light led to significantly (*p* < 0.05) increased lettuce shoot height at the seedling and baby-leaf growth stages regardless of PPFD (Figure 1A). Compared to the control (BRG), seedlings under B_30_ and B_60_ were 32% higher on average, and baby-leaf lettuce plants were about 30% (BBCH 14) and 32% (BBCH 18) higher. However, the leaf number was lower in EOD B-light-treated lettuce plants except in seedlings treated with a higher PPFD of B light at the end of the day (Figure 1B). No significant differences were found in the seedling and baby-leaf lettuce leaf width at BBCH *14*. However, baby-leaf lettuce at BBCH 18 formed significantly (on average, 21%) broader leaves regardless of B light PPFD (Figure 1C). EOD B light, on the other hand, led to significantly longer leaves of seedlings and baby-leaf lettuce. Compared to the BRG, seedlings under B_30_ had around 37% longer leaves, and under B_60_, about 24% longer. Baby-leaf lettuce at BBCH 14 had around 34% longer leaves under both EOD B light treatments, and at BBCH 18, about 26% (B_30_) and 30% (B_60_) (Figure 1D). However, no significant changes were found in the fresh weight (FW) and dry weight (DW) of lettuce plants except for the seedlings, which had around 60% higher FW under B_30_ and B_60_ PPFD and 58% higher DW under B_60_ compared to BRG (Figure 1E,F).

### 2.2. Photosynthetic Indices

**Figure 2:**
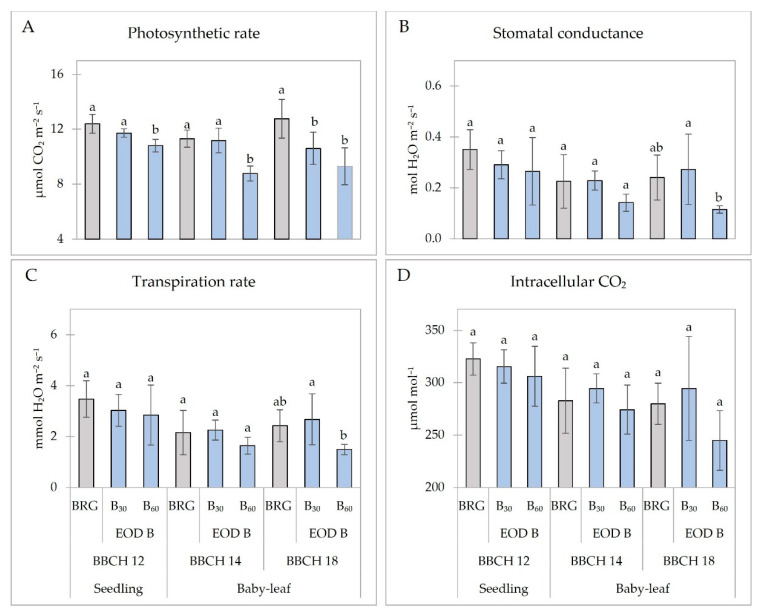
Photosynthetic indices ((**A**) photosynthetic rate; (**B**) stomatal conductance; (**C**) transpiration rate; (**D**) intracellular CO_2_) of lettuce plants at different development stages. BRG, 5% blue (peak = 450 nm), 85% red (peak = 660 nm), 10% green (peak = 530 nm) for 16 h d^−1^ (control; from 06:00 to 22:00); B_30_ and B_60_, PPFD of end-of-day lighting with blue (peak = 450 nm) at 30 and 60 µmol m^−2^ s^−1^, respectively; EOD, end-of-day lighting for 4 h d^−1^ (from 22:00 to 02:00). Data are means of two replications with three samples per replication (n = 6). Means with different letters are significantly different from the control treatment (BRG) at *p* < 0.05.

The additional B_60_ lighting significantly decreased the photosynthetic rate (Pr) of seedlings by 13% and of baby-leaf lettuce by 22% (BBCH 14) and 27% (BBCH 18) compared to BRG (Figure 2A). In addition, B_30_ decreased the Pr of baby-leaf lettuce by 17% at BBCH 18. No significant changes were found in the stomatal conductance (gs) of seedlings and baby leaf-lettuce at BBCH 14 (Figure 2B). However, baby-leaf lettuce had a significantly lower gs (by 52%) at BBCH 18. EOD B light did not influence the transpiration rate (Tr) of lettuce plants (Figure 2C). Additional EOD B lighting did not affect the intracellular CO_2_ concentration of lettuce, regardless of growth stage and B light PPFD (Figure 2D).

### 2.3. Spectral Reflectance Indices

**Figure 3:**
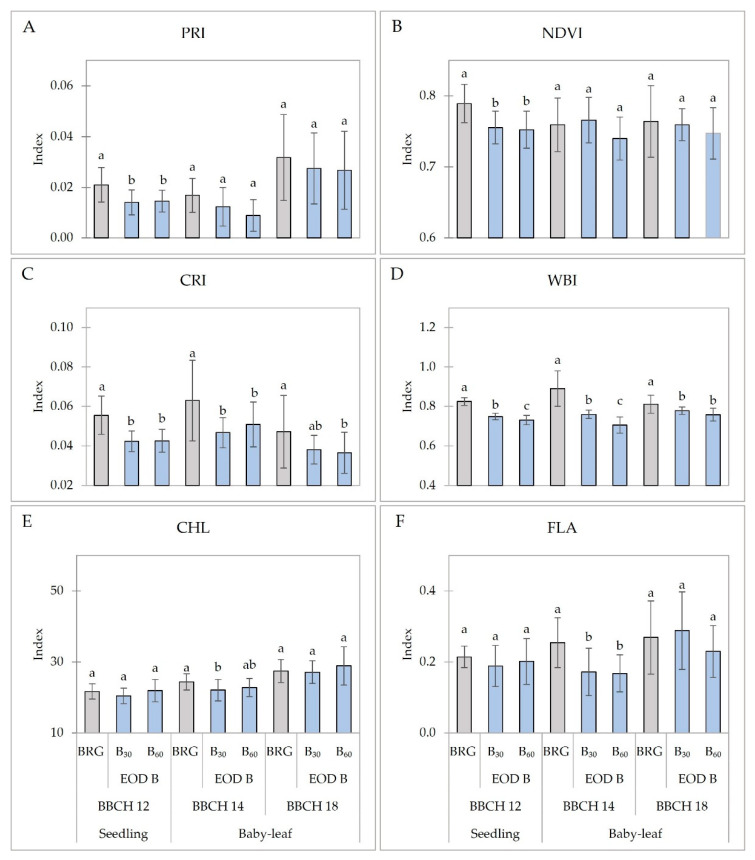
Spectral reflectance indices ((**A**) photochemical reflectance index, PRI; (**B**) normalized difference vegetation index, NDVI; (**C**) carotenoid reflectance index, CRI; (**D**) water band index, WBI; (**E**) chlorophyll index, CHL; (**F**) flavonol index, FLA) of lettuce plants at different development stages. BRG, 5% blue (peak = 450 nm), 85% red (peak = 660 nm), 10% green (peak = 530 nm) for 16 h d^−1^ (control; from 06:00 to 22:00); B_30_ and B_60_, PPFD of end-of-day lighting with blue (peak = 450 nm) at 30 and 60 µmol m^−2^ s^−1^, respectively; EOD, end-of-day lighting for 4 h d^−1^ (from 22:00 to 02:00). Data are means of two replications with ten samples per replication (n = 20). Means with different letters are significantly different from the control treatment (BRG) at *p* < 0.05. Indices on the ordinate axis are in relative units.

A significantly lower photochemical reflectance index (PRI) was determined for lettuce seedlings under B_30_ and B_60_ (33% and 29%, respectively) compared to that of BRG (Figure 3A). A similar trend was found in the normalized difference vegetation index (NDVI) of seedlings when EOD-B_30_ lighting slightly decreased the index by 4% and EOD-B_60_ by 5% compared to BRG (Figure 3B). No significant differences in the PRI and NDVI of baby-leaf lettuce were determined (Figure 3A,B). Both EOD B-light treatments decreased the carotenoid reflectance index (CRI) of lettuce regardless of growth stage. On average, about 24% lower CRI was determined in seedlings, and about 22% and 21% in baby-leaf lettuce at BBCH 14 and BBCH 18, respectively (Figure 3C). In addition, the EOD B light decreased the water band index (WBI) in lettuce plants. Compared to the BRG, lower WBI in seedlings and BBCH 14 and BBCH 18 baby-leaf lettuce (about 10%, 18%, and 5%, respectively) was determined (Figure 3D). No significant changes were found in the chlorophyll index (CHL) except for the lettuce at BBCH 14 under B_30_ when the about 10% lower CHL index was measured (Figure 3E). Similar results were observed in flavonol index (FLA) when EOD-B light decreased FLA by an average of 33% in lettuce at BBCH 14 (Figure 3F). 

### 2.4. Metabolic Response

**Figure 4:**
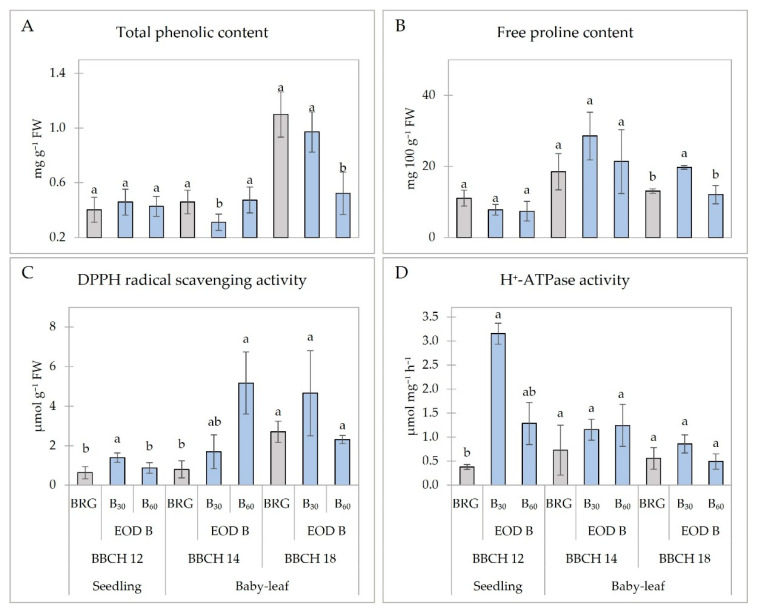
Metabolic response ((**A**) total phenolic content; (**B**) free proline content; (**C**) DPPH radical scavenging activity; (**D**) H^+^-ATPase activity) of lettuce plants at different development stages. BRG, 5% blue (peak = 450 nm), 85% red (peak = 660 nm), 10% green (peak = 530 nm) light for 16 h d^−1^ (Control; from 06:00 to 22:00); B_30_ and B_60_, PPFD of end-of-day lighting with blue light (peak = 450 nm) at 30 and 60 µmol m^−2^ s^−1^, respectively; EOD, end-of-day lighting for 4 h d^−1^ (from 22:00 to 02:00); PPFD, photosynthetic photon flux density; DPPH, 2,2-diphenyl-1-picrylhydrazyl. Data are means of two replications with three samples per replication (n = 6). Means with different letters are significantly different from the control treatment (BRG) at *p* < 0.05.

The additional EOD B light did not affect the total phenolic content (TPC) in lettuce seedlings. However, the B_30_ decreased TPC by 32% in lettuce at BBCH 14, and decreased TPC by 52% at BBCH 18 compared to BRG (Figure 4A). No differences in free proline content (PC) were observed except for lettuce at BBCH 18 under B_30,_ when about 51% significantly higher PC was measured (Figure 2B). The DPPH free radical scavenging activity was higher in lettuce plants treated with EOD B light. However, a significant increment was observed only in seedlings when B_30_ increased antioxidant activity by 21%, and in baby-leaf lettuce at BBCH 14 under B_60_ when antioxidant activity increased by more than six times compared to plants under BRG. The increased DPPH radical scavenging activity in lettuce plants at BBCH 18 was also measured, but the results were insignificant (Figure 4C). The highest H^+^-ATPase activity was determined in seedlings under B_30_, where it increased by about eight times compared to BRG. No significant changes in H^+^-ATPase activity in baby-leaf lettuce were determined (Figure 4D).

## 3. Discussion and Conclusions

In our study, the DLI of EOD treatments B_30_ and B_60_ increased by 0.43 and 0.86 mol m^−2^ d^−1^ or 3.7% and 7.4%, respectively, compared to the control BRG treatment. Such increment in DLI (especially 7.4%) could provoke the photoperiodic response of lettuce plants. However, we harvested lettuce at the seedling and baby-leaf stages, and could not observe the side effects of photoperiodic response, which occurs in more mature plants. We also considered lettuce to be a long-day and full-sun plant, and accepted that the changes in DLI during the experiments were low.

Blue light is involved in a wide range of plant processes such as phototropism, photomorphogenesis, stomatal opening, and leaf photosynthetic functioning [34]. The addition of blue light at the end of day provided the opportunity to evaluate its effect on plant growth and development. Kong et al. [35] suggested that promoted stem elongation by pure blue vs. red light might be one of the blue-light-mediated shade-avoidance responses under certain light levels (e.g., 50–100 μmol m^−2^ s^−1^ PPFD), with varying sensitivity levels among various plant species. Our results reveal that EOD B light could potentially increase plant growth indices such as shoot height or leaf length; however, it did not influence the leaf number (Figure 1). Seedlings had higher FW and DW. In addition, seedlings and baby-leaf lettuce at both BBCH phenological development stages under EOD B light formed longer leaves; however, the broader leaves were only of baby-leaf lettuce at BBCH 18. Moreover, longer leaves did not increase the FW and DW of baby-leaf lettuce. Such results indicate that the leaves of baby-leaf lettuce were fragile and easy to break. Our results partially agree with those of Chinchilla et al. [25], where no significant differences in leaf number of lettuce under EOD light, including B, were observed. According to the same study, lettuce grown under the low-intensity EOD B (50 µmol m^−2^ s^−1^) formed larger leaves compared to control (W light; 200 µmol m^−2^ s^−1^); however, the difference was insignificant. Contrary to our results, the lettuce plants had up to 18% higher FW and DW [25]. In the study by Yang et al. [36] low-intensity EOD B light at 10 µmol m^−2^ s^−1^ for 4 h influenced greater growth and the developmental parameters of kalanchoe (*Kalanchoe blossfeldiana*). EOD B light decreased the height, FW, and leaf area of dill plants at various growth stages [24]. The sole B light applied on mustard (*Brassica juncea*) microgreens increased growth indices such as FW, DW, and leaf area compared to dichromatic B and R light [37]. Li et al. [38] showed that EOD B light for 30 min led to the lowest FW and DW of Red Butter lettuce at 24 days after the treatment. However, EOD B light led to the significantly higher FW of Green Butter lettuce, indicating that the effects of EOD B were cultivar-dependent. Such contradictory results may reveal a plant phenomenon to promote growth elongation associated with other shade-avoidance responses. Furthermore, a combination of blue and red light results in synergetic effects in biomass accumulation. 

The study of Chinchilla et al. [25] showed that the Pr, gs, and Tr of lettuce were significantly higher when data were collected during the main photoperiod compared to predawn or EOD, which is most likely attributed to differences in PPFD at the different times (210 µmol m^−2^ s^−1^ for the main photoperiod vs. 50 µmol m^−2^ s^−1^ for predawn or EOD). In our study, the gas exchange data of lettuce treated with EOD B light were collected once during the main photoperiod (08:00 to 10:00 am). In general, EOD B negatively affected the gas exchange parameters of the investigated lettuce (Figure 2). The only increase in stomatal conductance (gs) was measured in baby-leaf lettuce at BBCH 18 under EOD B light of 60 µmol m^−2^ s^−1^ PPFD compared to BRG. The specific responses could be related to contradictory information from photoreceptors; signals from red light phytochromes and photosynthetic apparatus indicate the incidence of light, while the lack of a signal from blue-light receptors can be misinterpreted as darkness [39]. The results were contrary to those of Chinchilla et al. [1], where the gs and Tr of lettuce treated with EOD B light were significantly higher than those of the control (white LEDs). Only few specific processes in leaves were identified as quantitative blue light responses, such as chloroplast movement and stomatal conductance [40]. The blue-light enhancement effect on photosynthetic capacity appears to be greater when using combinations of red and blue light than that when broadband light is deficient in blue light [41]. However, Yang et al. [36] observed that significantly increased stomatal density and opening under low-intensity EOD B light positively affected CO_2_ absorption capacity, thus further improving the photosynthetic parameters of kalanchoe plants. 

Similar to photosynthetic parameters, the negative effects of EOD B light on the leaf spectral reflectance indices, such as photochemical reflectance index (PRI) and normalized difference vegetation index (NDVI), of seedlings were measured (Figure 3). However, EOD B light did not influence the PRI and NDVI of baby-leaf lettuce. the measured PRI and NDVI were in the range of green vegetation indices, according to Gamon et al. [42]. Moreover, EOD B light decreased the water band index (WBI) in lettuce plants, which is highly related to the growth and photosynthetic process of plants. The measured WBI indices in lettuce under the EOD B light were lower than those suggested by Penuelas et al. [43]. EOD B light treatments decreased the carotenoid reflectance index (CRI) of lettuce regardless of growth stage and PPFD compared to the BRG. The results agree with those Brazaitytė et al. [37], where monochromic B light decreased CRI, NDVI, WBI, and PRI in mustard microgreens. Li et al. [38] showed that EOD light does not affect carotenoid content in lettuce. No significant changes or lower chlorophyll (CHL) and flavonols (FLA) indices of lettuce were measured. The results partially agree with those of Chinchilla et al. [25], where no significant differences in the SPAD index in lettuce, which shows the relative content of chlorophylls, were found. Different EOD lights, including B, also had insignificant effects on most pigment contents and pigment ratios in two lettuce cultivars [38]. The sole B light decreased CHL and FLA in mustard microgreens [37].

The strong metabolic response of lettuce to EOD B light was observed (Figure 4). EOD B light negatively affected the accumulation of total phenolic compounds in baby-leaf lettuce that are responsible for free radical inhibition, peroxide decomposition, metal inactivation, or oxygen scavenging in biological systems to prevent oxidative burst [44,45]. No significant changes were observed by Li et al. in total phenolic content in two cultivars of lettuce during 24 days of EOD B light treatment for 30 min per day [38]. However, the strong antiradical activity of lettuce under EOD B light of 30 µmol m^−2^ s^−1^ was determined by DPPH free radical scavenging method. The results agree with those of Li et al. [38], where about 16% higher antiradical activity according to DPPH measurements was observed in Green Butter lettuce compared to white light. Moreover, the same trend of increased free proline content was found. Proline, an amino acid, plays a highly beneficial role as a metal chelator, an antioxidative defense or signaling molecule in plants exposed to various stress conditions, including light [46]. Plasma membrane H^+^-ATPase generates an H^+^ electrochemical gradient and provides a driving force for the uptake of various nutrients such as K^+^, nitrate, sulfate, sucrose, and amino acids across the plasma membrane in many cell types and tissues of the plant. Blue light activates H^+^-ATPase via phosphorylation [47]. H^+^-ATPase can be a mutual element for resistance mechanisms that are activated in various stress conditions caused by abiotic environmental factors [48,49]. 

Summarizing all obtained results, an increase in H^+^-ATPase activity in seedlings under EOD B light was observed, suggesting that the plants were under stress. EOD B light caused elevated shoot elongation in lettuce plants regardless of their growth stage. However, the leaf width increased only in more developed plants (BBCH 18). EOD B light negatively affected the development of new leaves and fresh weight, except for seedlings. The most photosynthetic and spectral leaf indices decreased when lettuce plants was treated with EOD B light, especially under the PPFD level of 60 µmol m^−2^ s^−1^. In addition, EOD B light induces metabolic and physiological changes in lettuce plants, such as DPPH free radical activity, free proline content, and H^+^-ATPase activity, so that the plant response to EOD B can be seen as a defensive response to unfavorable conditions. In future studies, more detailed research is needed to assess causality on morphological and biochemical changes in lettuce plants at different developmental stages in response to B light at the end of the day.

## 4. Materials and Methods

### 4.1. Growing Conditions

Lettuce (*Lactuca sativa,* Lobjoits Green Cos) (CN Seeds, UK) plants were grown in a walk-in and controlled-environment growth chamber at a constant 21 ± 2 °C temperature and relative air humidity of 60 ± 5%. The microclimate in the growth chamber was autonomously and independently controlled using a phytotron microclimate control system based on separate microcontrollers (AL-2-24MR-D, Mitsubishi Electric, Tokyo, Japan). The air temperature was measured with resistance temperature detectors (P-100; OMEGA Engineering Ltd., Norwalk, CT, USA), and data for these measurements were transmitted to the microcontrollers. The relative humidity and CO_2_ concentration were measured with capacitive sensors (CO2RT(-D); Regin, Kållered, Sweden) and controlled by additional humidifiers. Data were collected every minute, processed, and stored on the operator panel (E1000, Mitsubishi Electric, Tokyo, Japan).

Seeds of lettuce were sown into rock wool cubes (2.5 × 2.5 cm) and presoaked in deionized water with a pH of 4.4–4.5. Seeded cubes were placed in plastic trays and covered with agrotextile film. After four days, trays were uncovered, and deionized water was exchanged with a modified Hoagland nutrient solution containing the following average nutrient concentrations (mg L^−1^): N, 120; P, 20; K, 128; Ca, 72; Mg, 40; S, 53; Fe, 4; Mn, 0.08; Cu, 0.08; B, 0.16; Zn, 0.8. The pH was 5.5–6.5, and the electrical conductivity (EC) was 0.13–0.17 S m^−1^ (GroLine HI9814, Hanna Instruments, Woonsocket, RI, USA). 

### 4.2. Lighting Treatments

Plants were grown under controllable lighting fixture Heliospectra RX30 (Gothenburg, Sweden) consisting of 5% blue (B, peak = 450 nm), 10% green (G, peak = 530 nm), and 85% red (R, peak = 660 nm) light-emitting diodes (LEDs) at the photosynthetic photon flux density of 200 µmol m^−2^ s^−1^ for 16 h d^−1^ (treatment code BRG, control). The daily light integral (DLI) was 11.52 mol m^−2^ d^−1^. For the end-of-day (EOD) treatments, lettuce samples were additionally illuminated with 100% of B light at 30 and 60 µmol m^−2^ s^−1^ PPFD for 4 h (treatment codes B_30_ and B_60_, respectively). The DLI of additional EOD B light was 0.43 mol m^−2^ d^−1^ for B_30_ and 0.86 mol m^−2^ d^−1^ for B_60_ treatment (Table 1). The photon distributions of all lighting treatments were measured using a portable spectroradiometer (WaveGo, Wave Illumination, Oxford, Oxfordshire, UK) at the tray surface level. Lettuce samples were grown until the three development stages: seedling (8 days, BBCH 12) and baby-leaf (15 and 25 days, accordingly BBCH 14 and BBCH 18). The experiments were repeated twice.

### 4.3. Growth Measurements

Biometric measurements were conducted on 10 seedlings (BBCH 12) and baby-leaf (BBCH 14 and BBCH 18) lettuce from each lighting treatment and experimental replication. Each plant was cut from the rock wool cube, and shoot fresh weight (FW, g) and dry weight (DW, g) were measured using an analytical balance (Mettler Toledo AG64, Columbus, OH, USA). The leaf length (cm) and width (cm) of the second true leaf of seedlings and the third fully expanded leaf of baby-leaf lettuce were measured, and leaves (when >2 cm) were counted. Shoots were dried in an oven (Venticell 222, MBT, Brno-Zábrdovice, Czech Republic) at 70 °C for 48 h before DW measurements. 

### 4.4. Measurements of Photosynthesis

The photosynthetic rate (Pr, µmol CO_2_ m^−2^ s^−1^), transpiration rate (Tr, mmol H_2_O m^−2^ s^−1^), stomatal conductance (gs, mol H_2_O m^−2^ s^−1^), and intracellular CO_2_ concentration (µmol m^−1^) were measured using portable photosynthesis system LI-COR 6400XT (LI-COR, Inc., Lincoln, NE, USA). The leaf chamber conditions were set to be 21 °C, CO_2_ concentration of 400 µmol mol^−1^, and 60% relative humidity. Artificial irradiation in the leaf chamber was supplied from 665 and 470 nm LED sources, PPFD at 1000 µmol m^−2^ s^−1^. Photosynthesis was measured from 8:00 to 10:00 a.m. 

### 4.5. Nondestructive Measurements of Spectral Indices

Nondestructive measurements of leaf chlorophyll (CHL) and flavonol (FLA) indices were performed using a Dualex 4 Scientific^®^ (FORCE-A, Orsay, France) meter.

Spectral reflectance indices were measured using a leaf spectrometer CI-710 (CID Bio-Science, Inc., Camas, WA, USAsec) from 8:00 to 10:00 am. Reflection spectra obtained from the leaves were used to calculate the photochemical reflectance index (PRI), which shows changes in the xanthophyll cycle [42], using the following formula:

(1) $$\mathrm{PRI}=\frac{R531-R570}{R531+R570};$$

The normalized difference vegetation index (NDVI), which shows changes in biomass content, was calculated with:

(2) $$\mathrm{NDVI}=\frac{R800-R680}{R800+R680};$$

The carotenoid reflectance index (CRI), which shows changes in the carotenoids to chlorophyll ratio, was calculated with:

(3) $$\mathrm{CRI}=\frac{1}{R510}-\frac{1}{R700};$$

The water band index (WBI), which shows canopy water content [43], was calculated with:

(4) $$\mathrm{WBI}=\frac{W900}{W970},$$

where W970, W900, R800, R680, R570, R531, and R510 represent leaf reflectance integrated over a 10 nm wavelength band centred on 970, 900, 800, 680, 570, 531, and 510, respectively.

### 4.6. Determination of Total Phenolic Content

For the determination of total phenolic content (TPC), 500 mg of fresh plant material was frozen in liquid nitrogen, homogenized with 5 mL of 80% ice-cold methanol (SIGMA-ALDRICH Chemie GmbH), and transferred to a 15 mL polypropylene conical centrifuge tube (Falcon, Thermo Fisher Scientific Inc.). The extract was incubated at 4 °C for 24 h. After incubation, the samples were centrifuged for 15 min at 3000 rpm and filtered through Whatman Grade 1 qualitative filter paper. The TPC of lettuce was determined spectrophotometrically, with slight modifications, according to Ainsworth and Gillespie [50]. First, 100 µL of the extract was diluted with 200 µL of 10% (vol/vol) Folin and Ciocalteu’s phenol reagent (SIGMA-ALDRICH Chemie GmbH) and vorticed thoroughly. Then, 800 µL of 700 mM sodium carbonate (SIGMA-ALDRICH Chemie GmbH) was added. After 20 min, the absorbance of the samples was measured using a spectrophotometer (M501, Spectronic Camspec Ltd., Leeds, UK) at 765 nm. The TPC in fresh plant tissues was calculated using a standard curve of gallic acid (*R^2^* > 0.95). Data are presented as the mean of three analytical measurements of TPC in mg g^−1^ FW.

### 4.7. Evaluation of DPPH Free Radical Scavenging Activity

The same extracts from TPC measurements were used to evaluate 2-diphenyl-1-picrylhydrazyl (DPPH, SIGMA-ALDRICH Chemie GmbH) free radical scavenging activity. The antiradical activity was evaluated according to the spectrophotometric DPPH scavenging activity method [51,52] with modifications. Then, 100 µL of 80% methanol extracts used for the TPC assay was diluted with 1 mL of 60 µM DPPH solution. Absorbance at 515 nm was measured after 16 min (M501, Spectronic Camspec Ltd., Leeds, UK). The ability of plant extracts to scavenge DPPH free radicals was calculated using the DPPH solution as a blank. Data are presented as the mean of three analytical samples to scavenge DPPH free radicals in µmol g^−1^ FW.

### 4.8. Determination of Free Proline Content 

The acidified ninhydrin colour reaction was used to determine proline content [53,54]. Each 500 g lettuce sample was ground in a prechilled mortar for 2 min, and proline with 10 mL of 3% sulphosalicylic acid (Roth) was extracted at 4 °C for 24 h. The extracts were then centrifuged at 700× *g* (centrifuge MPW-351 R, Poland) for 20 min. The supernatant, acetic acid, and acidified ninhydrin were mixed at a 1:1:1 ratio, and heated for 1 h at 100 °C in a BLOCKTHER-MOSTAT BT 200 (Kleinfeld Labortechnik, Gehrden, Germany). The reaction was stopped in an ice bath and cooled for 15 min. The resulting chromophore was extracted with toluene (Roth), vortexed vigorously, and incubated in the dark for 1 h. Absorbance was measured with a Rainbow microplate reader (SLT Lab-Instruments, Rendsburg-Eckernförde, Germany) at 520 nm. Proline content was estimated using a calibration L-proline (Roth) curve. Results are expressed as mg proline per 100 g FW. Calculations were performed using the SLT programme (SLT Labinstruments, Salzburg, Austria). Results are expressed as µmol of proline per g of fresh mass.

### 4.9. Determination of H^+^-ATPase Activity

H^+^-ATPase activity in the membrane samples was assessed at 37 °C (0.5 h) by measuring the release of inorganic phosphate (Pi), which accumulates as a result of ATP hydrolysis [53,54]. Briefly, the plant material was gently homogenised in Tris-HCl buffer (pH 7.8). Then, differential centrifugation was used to separate the membrane fraction: 5 min at 4500× *g*, and 20 min at 18,000× *g* (centrifuge MPW-351 R, Poland). The resulting supernatant was centrifuged for 1 h at 92,200× *g* (Thermo Scientific Sorvall WX 100 Ultra, Waltham, MA, USA). The precipitate that formed was resuspended in a glass potter. The Bradford assay [55] was used to estimate the protein content in the membrane fraction. Ammonium molybdate and stannous chloride were used for the Pi color reaction, of which the absorbance was read at 750 nm. H^+^-ATPase activity is expressed as µmol Pi produced per hour per mg protein. 

### 4.10. Statistical Analysis

Statistical analysis was performed using Microsoft Excel Version 2206, and Addinsoft XLSTAT 2022 statistical and data analysis (Long Island, NY, USA). One-way analysis of variance followed by Tukey’s honestly significant difference test (*p* < 0.05) for multiple comparisons was used to evaluate the differences between means of measurements.

## Figures and Tables

**Table 1:** Photon distribution of sole-source lighting for lighting treatments used in experiments.

Lighting Treatment	16 h			EOD 4 h	DLI, mol m^−2^ d^−1^
	B, 450 nm	G, 530 nm	R, 660 nm	B, 450 nm	
	PPFD, µmol m^−2^ s^−1^				
BRG (Control)	10	20	170	0	11.52
B_30_	10	20	170	30	11.95
B_60_	10	20	170	60	12.38

BRG, blue (peak = 450 nm), red (peak = 660 nm), green (peak = 530 nm) light for 16 h d^−1^ (control); B_30_ and B_60_, PPFD of end-of-day lighting with blue light (peak = 450 nm) at 30 and 60 µmol m^−2^ s^−1^, respectively, for 4 h d^−1^; PPFD, photosynthetic photon flux density; DLI, daily light integral.

## Data Availability

Not applicable.
